# A review of ureteral injuries after external trauma

**DOI:** 10.1186/1757-7241-18-6

**Published:** 2010-02-03

**Authors:** Bruno MT Pereira, Michael P Ogilvie, Juan Carlos Gomez-Rodriguez, Mark L Ryan, Diego Peña, Antonio C Marttos, Louis R Pizano, Mark G McKenney

**Affiliations:** 1 DeWitt Daughtry Family Department of Surgery, Leonard M. Miller School of Medicine, University of Miami/Jackson Memorial Hospital, Ryder Trauma Center, Miami, FL, USA; 2 Universidad Militar Nueva Granada, Hospital Militar Central, Servicio de Cirurgia General, Bogotá, DC, Colombia

## Abstract

**Introduction:**

Ureteral trauma is rare, accounting for less than 1% of all urologic traumas. However, a missed ureteral injury can result in significant morbidity and mortality. The purpose of this article is to review the literature since 1961 with the primary objective to present the largest medical literature review, to date, regarding ureteral trauma. Several anatomic and physiologic considerations are paramount regarding ureteral injuries management.

**Literature review:**

Eighty-one articles pertaining to traumatic ureteral injuries were reviewed. Data from these studies were compiled and analyzed. The majority of the study population was young males. The proximal ureter was the most frequently injured portion. Associated injuries were present in 90.4% of patients. Admission urinalysis demonstrated hematuria in only 44.4% patients. Intravenous ureterogram (IVU) failed to diagnose ureteral injuries either upon admission or in the operating room in 42.8% of cases. Ureteroureterostomy, with or without indwelling stent, was the surgical procedure of choice for both trauma surgeons and urologists (59%). Complications occurred in 36.2% of cases. The mortality rate was 17%.

**Conclusion:**

The mechanism for ureteral injuries in adults is more commonly penetrating than blunt. The upper third of the ureter is more often injured than the middle and lower thirds. Associated injuries are frequently present. CT scan and retrograde pyelography accurately identify ureteral injuries when performed together. Ureteroureterostomy, with or without indwelling stent, is the surgical procedure of choice of both trauma surgeons and urologists alike. Delay in diagnosis is correlated with a poor prognosis.

## Introduction

### Background

The proper management of a trauma victim is an increasingly relevant topic of discussion due to international warfare and the growing domestic incidence of traumatic injury. According to the Center for Disease Control and Prevention (CDC), trauma is the leading cause of death in children and young adults and overall is the fifth leading cause of death in the United States [1]. The World Health Organization classifies trauma as the 9^th ^leading cause of death worldwide [2,3].

Ureteral trauma was first reported in 1868 by Alfred Poland when he described the first case of disruption from blunt trauma [4]. The patient was a 33-year-old woman who died 6 days after being pinned between a platform and a railway carriage. At autopsy, in addition to many other injuries, the right ureter was avulsed below the renal pelvis [5]. Henry Morris described the first ureteral procedure in 1904, when he performed an ureterectomy on a 30-year-old male who "fell from his van catching one of the wheels across his right loin" [6]. In both cases, the ureteral injury was missed upon admission. Kirchner reported the first bilateral ureteral injury and repair, secondary to a single low-velocity penetrating missile, in 1981 [7].

Genitourinary (GU) trauma is often overlooked in the setting of acute trauma due to immediate, life-threatening injuries taking precedence, but accounts for roughly 10% of all injuries seen in the emergency room. Ureteral trauma is uncommon, accounting for less than 1% of all urologic trauma [8]. However, a missed ureteral injury can result in significant morbidity and mortality.

The rationale for this article is to review the literature since Zufall et al published the first indexed series on ureteral trauma in 1961 [9], with the primary objective to present the largest review of the literature concerning ureteral trauma. This article summarizes the background of ureteral traumatic injuries, provides a review of the surgical approaches to their treatment and proposes an updated management algorithm.

### Anatomic and physiologic considerations

**Figure 1:**
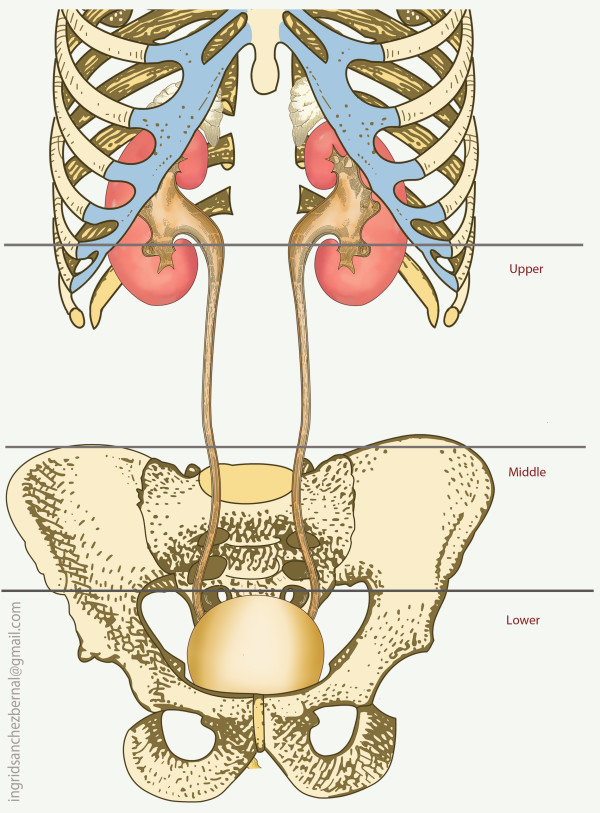
**Anatomic division of the ureter**.

Ureteral injuries (UI) due to trauma are rare as the ureter is well protected in the retroperitoneum by the bony pelvis, psoas muscles and vertebrae [10,11]. The left ureteropelvic junction is posterior to the pancreas and ligament of Treitz. The inferior mesenteric artery and sigmoidal vessels cross in front of the left ureter at its inferior pole. On the right side, the ureter lies posterior to the duodenum and just lateral to the inferior vena cava, with the right colic and ileocolic vessels crossing in front. Due to this protection, injuries to the ureter are typically accompanied by significant collateral damage and management is dictated by the severity of associated injuries [10-13]. Anatomically, the ureter is 22 to 30 cm in length and is divided into three portions: the proximal ureter (upper) is the segment that extends from the ureteropelvic junction to the area where the ureter crosses the sacroiliac joint, the middle ureter courses over the bony pelvis and iliac vessels, and the pelvic or distal ureter (lower) extends from the iliac vessels to the bladder (Fig. 1). The terminal portion of the ureter may be subdivided further into the juxtavesical, intramural, and submucosal portions. The surgeon must pay special attention to the gonadal and iliac vessels, as they cross the ureter at the posterior and anterior levels respectively, descending into the pelvis.

The ureter's blood supply comes from the ureteral artery, which runs longitudinally along the ureter and lacks collateral flow in 80% of patients. The upper third of the ureteral artery is supplied by the aorta and renal artery, while branches of the iliac, lumbar and vesicular arteries supply the middle and lower thirds of the ureter. In the abdomen the blood supply is medial, while in the pelvis the blood supply is lateral with the richest blood supply to the pelvic ureter. From a surgical standpoint, knowledge of the vascular supply to the ureter is crucial prior to any manipulation and subsequent repair. This tenuous blood supply must be considered when dealing with complex repairs of significant injuries and strict adherence to the principles of ureteral repair can prevent complications such as leak, renal injury and in some cases, death [14-17].

Histologically, the ureter consists of three distinct layers. The first is an inner mucosal layer of transitional epithelium covered by lamina propria. The inner layer produces mucosal secretions to protect itself from urine. The second or middle layer is muscular and consists of both longitudinal and circular layers of smooth muscle, which help propel urine forward by peristalsis. The outer (adventitial) layer consists of areolar connective tissue and contains nerves, blood vessels and lymphatic vessels.

No continuous lymph channels extend from the kidney to the bladder. Lymphatic drainage from the ureter drains to regional lymph nodes including the common iliac, external iliac and hypogastric lymph nodes.

The ureter is a dynamic organ rather than a simple conduit through which urine flows. It conducts urine from the renal papillae to the ureteral orifices in the bladder irrespective of the spatial orientation of the body. However, when the urinary transport system is disturbed, gravity may influence directional flow [18]. Three major functions are attributed to the renal pelvis and ureters: absorption, dynamics, and tonus. Absorption is minimal and unaffected by repair of the ureter and its consequent function. The dynamics reflect the synchronous and progressive contractile movement of the ureter away from the ureteropelvic junction (UPJ) to the ureter-vesical orifice, produced by the intrinsic automaticity of the ureteral musculature [14,18]. Tonus of the ureter is the degree of contraction that the ureteral wall assumes for a given rate and volume of urinary output. Tonus initiates detrusor action at a certain volume, thus perpetrating the cyclical undulations. When a ureter is damaged by penetrating or blunt trauma, peristalsis beyond the injury ceases. Tonus is decreased in the ureter, proximal to the injury, due to stretching from the increased volume of urine in this segment. This increased volume of urine is the result of detrusor action being halted at the damaged (inert) segment of the ureter [19]. Thus, urine volume, diuresis and distention are the main modulators of peristalsis along with the sympathetic and parasympathetic nervous system; however, prostaglandins and tachykinins also play a role.

### Wound Ballistics

Ballistics is the study of the motion of projectiles in flight and wound ballistics is the study of the motion of missiles within the body and their wounding capacity. The trauma surgeon must be knowledgeable in both ballistics and wound ballistics in order to better understand the mechanism of injury.

The ureter may be injured by penetrating (i.e. gunshot or stab wounds) or blunt trauma. The relative predominance of ureteral injury associated with gunshot wounds is reflected in the characteristics of the permanent cavity trajectory of the bullet and the missile blast injury (temporary cavity). The bullet can damage the ureter via direct transection or the blast injury caused by the missile may disrupt the intramural blood supply, resulting in ureteral necrosis. Fortunately, fewer than 3% of gunshot injuries involve the ureters [11].

The powerful stretch due to blast effect (temporary cavity) caused by low velocity missiles over the ureter and adjacent tissues may not be immediately apparent during laparotomy or by extravasation of contrast during imaging. However, the blast contusion can seriously damage the small ureteral blood vessels producing thrombosis and ischemia, which eventually results in delayed necrosis and complications (i.e. urine leakage and ureteral fistula). Therefore, the surgeon must be aware that the integrity of the ureter may be in jeopardy for several days post-injury [7,16,20-25].

As the incidence of trauma has increased over the years, so too has the incidence of ureteral injury. Additionally, as the power of the weapons utilized increases, the characteristics of penetrating trauma continue to evolve. High velocity missile wounds are much more commonplace and are a challenging entity for trauma surgeons. Gunshot wounds are mainly low velocity and typically create only localized damage. In contrast, the significant kinetic energy of high velocity missiles result in extensive damage to the surrounding tissue creating temporary cavities in the order of 30 to 40 times larger than the size of the permanent cavity. This extremely high pressure can cause irreversible damage to adjacent tissues and it is imperative that the trauma surgeon be aware of these devastating effects [25,26].

## Literature review

### Methods

The following electronic databases were used to identify publications for this review: Bireme/Lilacs (Latin America and Caribbean Center on Health Science Information, Pan American Health Organization - Virtual Health Library), Cochrane Library (Injuries Group's), Embase, Medline, Pubmed and Springer Link.

Key words used: "ureter", "ureteral", "traumatic", "trauma", and "injury".

Eighty-one articles were initially identified. Publications were excluded if they did not mention data on demographics, type of trauma or clinical/surgical approach. Articles were also excluded if they were not written in English, Spanish or Portuguese. Overall, only four articles were excluded with the remaining articles compiled into Additional file 1 - Table S1.

The medical literature review table (Additional file 1 - Table S1) is organized by year of publication in descending order. Authors, study design, objectives, incidence (demographics, type of injury, ureter injured portion), admission diagnostics (urine analysis, IVU, CT scan, RPG, intraoperative diagnosis), surgical technique and complications (early and late) were compiled. Of note, missed injuries were considered a late complication.

### Results

Literature search identified 77 retrospective reviews with a total of 1021 patients. All articles were classified as level of evidence (LOE) 3 or 4 (retrospective studies and case reports).

Of all compiled patients, 83.4% (± 28.5) were males and the average age was 23.2 years old (± 12.1), reflecting young male predominance in violent trauma.

The majority of ureteral injuries (61.1% ± 45.7) were caused by a penetrating mechanism. Proximal ureteral injury occurred at a rate of 59.7% (± 37), while mid and distal injuries occurred 25.6% (± 30.4) and 20.8% (± 24.4) of the time, respectively.

Associated injuries were present in 90.4% (± 26.2) of patients, indicating that ureteral injuries often occurs as part of a myriad of problems associated with significant trauma. Small and/or large bowel injuries were most commonly involved in conjunction with ureteral trauma (96% ± 21.5).

When performed, admission urinalysis demonstrated hematuria in only 44.4% (± 36.3) of patients. Intravenous ureterogram (IVU) failed to diagnose ureteral injuries either upon admission or in the operating room in 42.8% (± 38) of cases. However, when a CT scan and retrograde pyelogram were performed together they were able to accurately identify ureteral injuries - in an early or delayed setting, 88.3% (± 28.2) of the time. Intraoperative diagnoses were made in 62% (± 38.8) of cases.

Ureteroureterostomy, with or without indwelling stent, was the surgical procedure of choice of both trauma and urology surgeons (59% ± 34).

Complications occurred in 36.2% (± 34) of cases, including retroperitoneal abscesses, infected urinomas and fistulas; these were usually secondary to a delay in diagnosis. Missed ureteral injuries were reported in 38.2% (± 39.5) of the cases. The associated mortality rate of the study population was 17%, although the contribution from the ureteral injury is difficult to quantify.

## Diagnosis and management

In diagnosing ureteral injuries from trauma, the most important factor is a high index of suspicion [27]. Typically there are no classic signs or symptoms for ureteral injuries, but should be suspected in all cases of penetrating abdominal injury and in cases of blunt deceleration trauma, particularly in children in whom the kidney and renal pelvis can be torn from the ureter, secondary to their hyper-extensible vertebral column [10,11,28]. Although some authors advocate that hematuria is the hallmark of any GU lesion, it is present in only half (43%) of those with UI, indicating that hematuria is not a sensitive indicator of ureteral trauma [10,13,17,28-30]. Therefore, any patient that presents with gross hematuria, flank pain or ecchymosis should undergo more extensive investigation [16,20,28,29].

Unfortunately, there is no imaging modality best suited to diagnose acute ureteral injury. The use of ultrasound has gained widespread use in trauma but has proven unreliable in evaluating ureteral injuries, particularly because of their small caliber and retroperitoneal location. According to the European Association of Urology guidelines, computed tomography (CT) and an intra-operative single-shot intravenous pyelogram (IVP) are the most useful diagnostic tools, but some authors have argued against the reliability of single-shot IVP [10,11,17,30-34]. Complete IVP (which includes all excretory phases) has proven a reliable study in the stable trauma patient for diagnosing ureteral trauma but is often impractical given the precarious nature of most trauma victims [35-39]. Retrograde pyelography is believed to be the most accurate method of diagnosis but is not feasible in hemodynamically unstable patients. For the stable patient who can undergo a CT scan, delayed excretory phase images have the benefit of not only showing extravasation of contrast media from the ureteral injury, which may be subtle, but can also illustrate accompanying lesions, particularly involving the kidney [12,30,32,33]. In the delayed setting, a CT may also diagnose missed ureteral injuries (i.e. ascites, urinomas, hydronephrosis and contrast extravasation).

The American Association for the Surgery of Trauma (AAST) created a grading scale of ureteral injuries (UI) (Table 1) [40] and surgical management has been shown to be highly dependent on the AAST grade, site of the injury, associated injuries and whether the ureteral injury is diagnosed in the acute or delayed setting [10-13,17,19].

**Table 1:** AAST Classification for Ureteral Injuries (adapted)

Grade	Description of Injury
I	Hematoma only
II	Laceration < 50% of circumference
III	Laceration > 50% of circumference
IV	Complete tear < 2 cm of devascularization
V	Complete tear > 2 cm of devascularization

**Figure 2:**
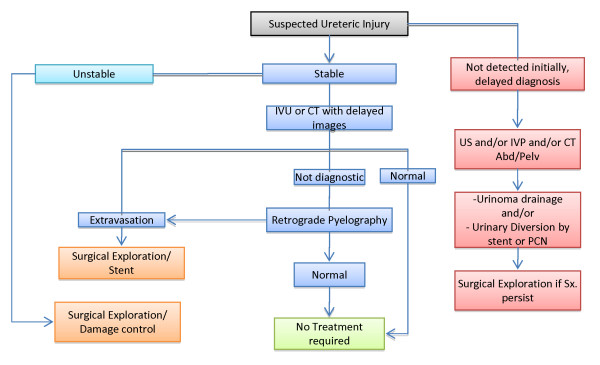
**Ureteral trauma algorithm**.

The primary objective of ureteral repair is preservation of renal function. Hence, the most important factor in the management of these injuries is to maintain drainage of urine from the kidney and to prevent the formation of urinoma and abscess [19]. The algorithm for external ureteric trauma is shown in Fig 2[30].

Injuries identified in the early phase may be surgically repaired over a stent using fine absorbable sutures, assuming a tension free, healthy tissue anastomosis can be achieved. Large ureteric injuries present a significant problem, especially in the upper and mid zones, as they may require significant reconstruction [13,17,19,36,41-46]. Successful repair methods for acute ureteric injuries are based on certain principles: ureteric debridement and careful mobilization, spatulated, tension-free, water-tight anastomosis over a stent (5-0 absorbable suture under magnification), isolation of the ureteric repair from associated injuries and adequate drainage of the retroperitoneum [19,30,36,42,43].

Some authors oppose the use of indwelling stents in the setting of ureteral trauma, citing such potential problems as obstruction, stricture formation, inflammation from the foreign body, stent migration and patient discomfort, however, this is not supported by the current surgical literature [36,47]. Other authors have argued that the benefits of the ureteral stent in the management of this injury far outweigh the potential risks and advocate use of a stent, especially in the setting of high-velocity gunshot wounds [12,37,38,41,48-51].

**Figure 3:**
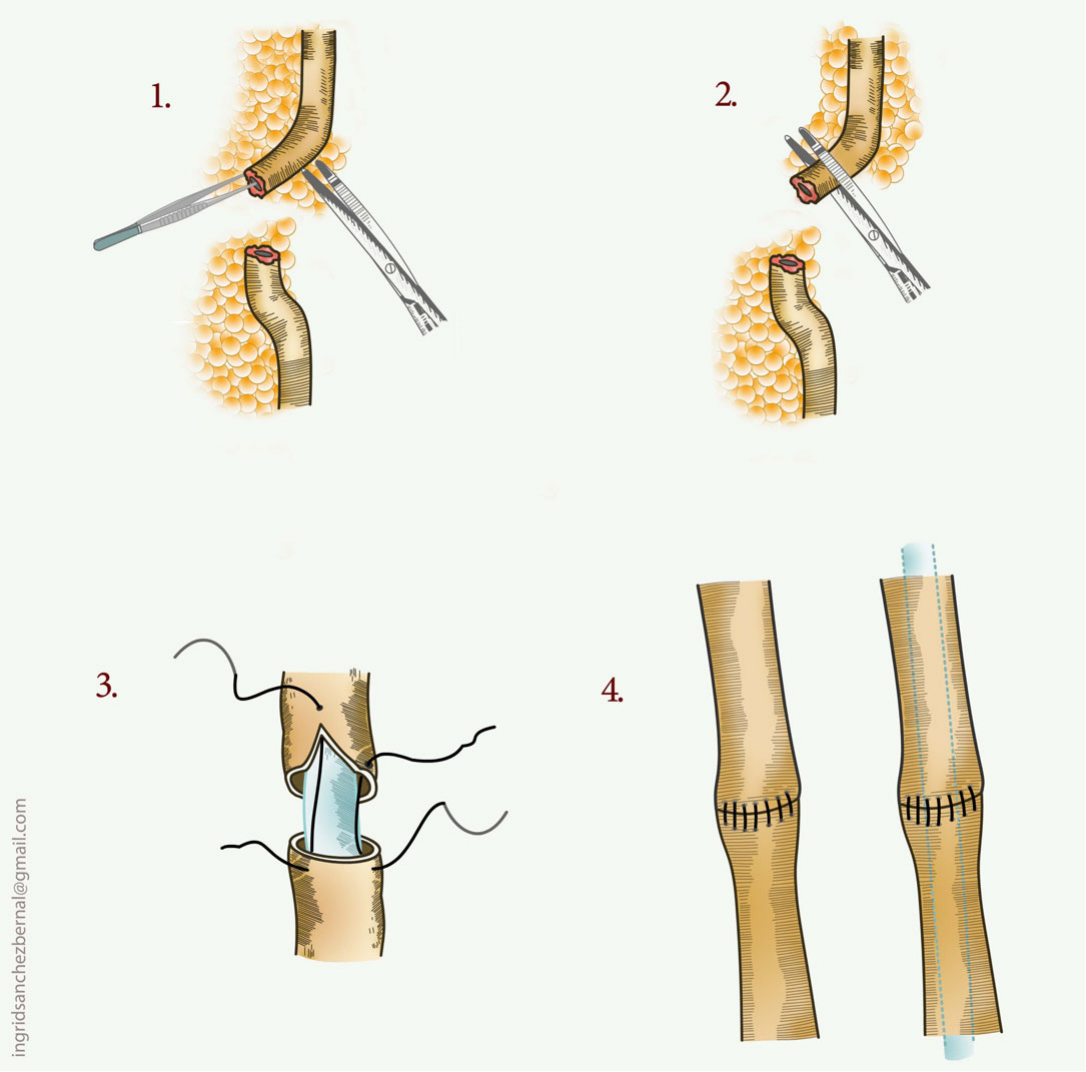
**Uretero-ureterostomy**.

**Figure 4:**
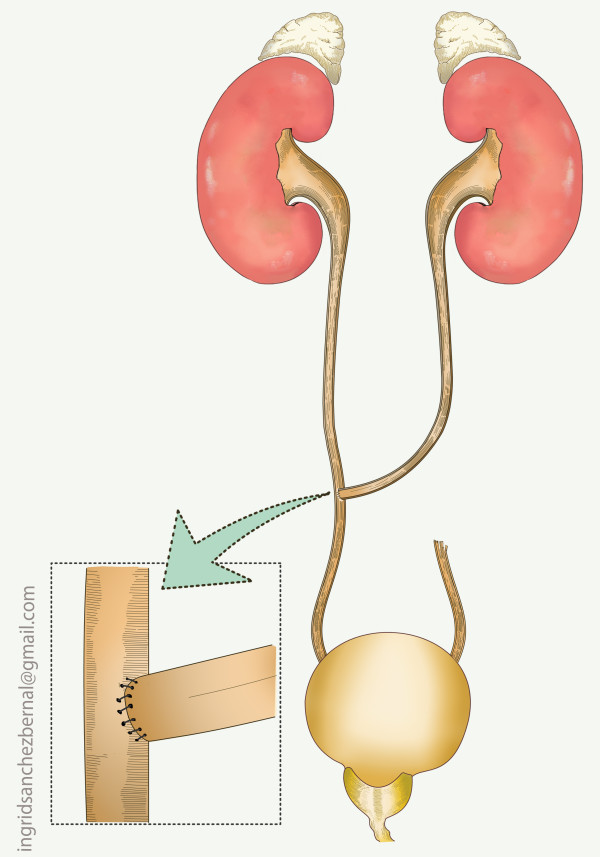
**Transureteral ureterostomy**.

**Figure 5:**
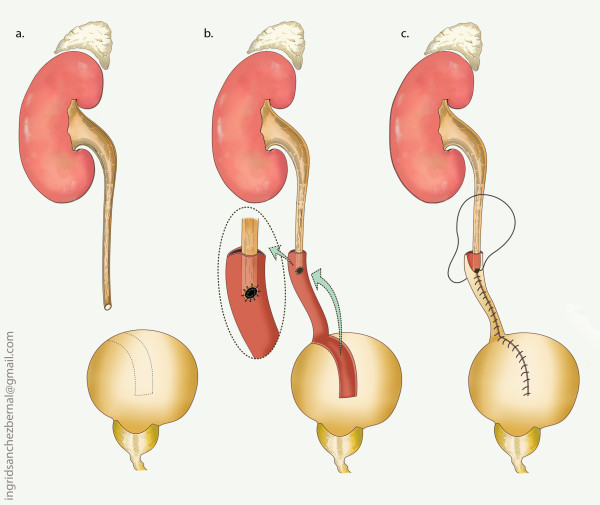
**Boari flap**.

**Figure 6:**
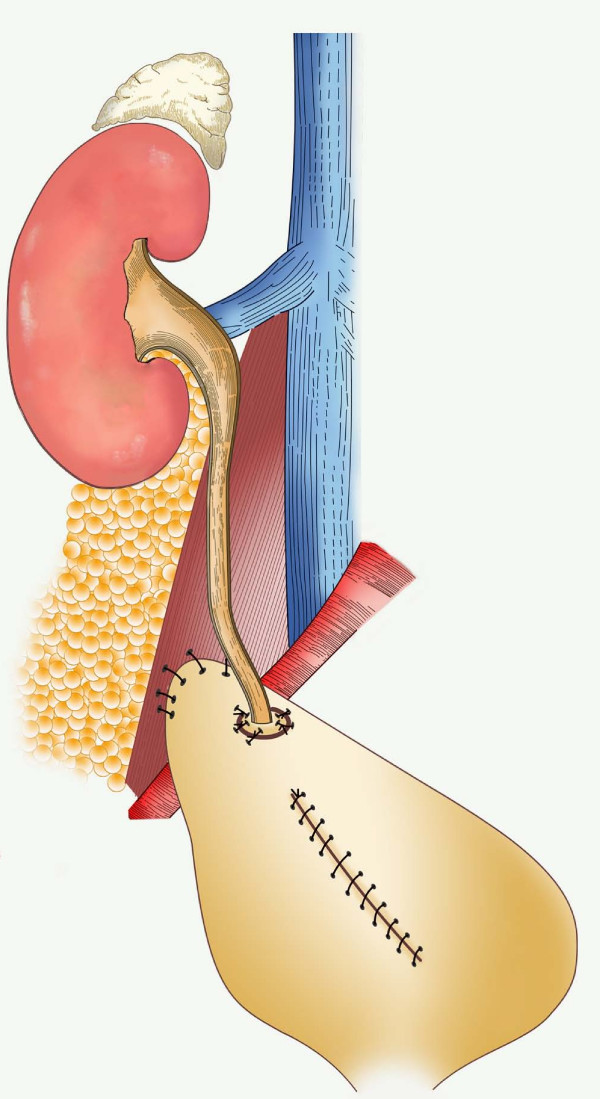
**Psoas Hitch**.

The pertinent reconstructive options, based on location are presented in table 2 (Figs 3, 4, 5, 6).

**Table 2:** Pertinent reconstructive options, based on location

Upper third	Uretero-ureterostomy (Fig. 3)
	Ureteropyelostomy
Middle third	Uretero-ureterostomy
	Transuretero-ureterostomy (Fig. 4)
	Anterior wall bladder flap (Boari) (Fig. 5)

Lower third	Ureteroneocystostomy (direct reimplantation)
	Ureteroneocystostomy (psoas hitch) (Fig. 6)

Failure of prompt diagnosis can lead to several complications including renal failure, sepsis and death. More common complications include the formation of urinomas, periureteral abscess, fistulas and strictures. However, these complications are readily preventable and can occur less than 5% of the time with proper stenting and/or placement of a nephrostomy tube [10,17,43]. Surgical repair is typically recommended for delayed complications such as fistulas and strictures. The early diagnosis of ureteral injury is extremely important and directly related to the patient's prognosis [8,24,25,30,41,52-62]. In the articles reviewed, late diagnoses including missed injuries were correlated with higher rates of morbidity and mortality.

Other factors that might confound the diagnosis of ureteral trauma are: pre-existing renal pathology, associated injuries, acute and chronic renal failure, arteriovenous fistula and renovascular hypertension [45].

### Special Considerations

In the event of a complete loss of the ureter, the various surgical options have been well documented; these include an appendiceal interposition (children - delayed), an ileal segment interposition (delayed), or autotransplantation [63-69].

The vermiform appendix has been used as a conduit in some cases (adults or children) and is another surgical option for complete ureteric loss in the non-acute trauma setting. The appendix is similar to the ureter in caliber and mucosal surface area. Additionally, there is no significant absorption of sodium chloride or urea and hence electrolyte disturbances are not seen (as has been described with ileal interposition grafts). The reported disadvantages of using the appendix are stenosis, anastomotic dehiscence, fistula formation and inadequate length, which may exclude its use in significant ureteral loss. Anastomotic breakdown is reported to have a higher incidence in isoperistaltic interposition. Antiperistaltic interposition is therefore recommended to theoretically reduce torsion of the mesoappendix and thus prevent further vascular compromise [63-69].

An ileal interposition, much like the appendiceal interposition, is not performed in the acute setting due to the need for bowel preparation. Despite reported success rates of up to 81%, several authors condemn this approach for its high complication rate [29,30,61,70-75]. Reported complications include urosepsis, vesicoileal reflux, obstruction, excess mucus formation resulting in obstruction secondary to the formation of mucus plugs, urolithiasis and electrolyte disturbances, most commonly in the form of metabolic acidosis [70-75].

Autotransplantation involves relocating the ipsilateral kidney to the pelvis; the renal artery and vein are then anastomosed to the iliac vessels and the healthy ureter or renal pelvis is anastomosed to the bladder. Autotransplantation is less desirable than use of the appendiceal or ileal conduit for massive ureteral loss due to its complex nature.

Fibrin sealant is being applied more often in various surgical fields, including urology. It has proven to be safe in trauma and to reinforce ureteral anastomosis [76-79]. Fibrin glue has not been shown to have adverse effects in rabbit models [80].

## Conclusion

Ureteral injuries (UI) due to trauma are unusual. However, failure to take this type of injury into consideration can have dire consequences, as complications from missed injuries are a cause of severe morbidity and mortality. This is the largest review of the literature regarding traumatic ureteral injuries and from this several things are evident. First, penetrating injuries are more common than blunt ureteral injuries in adults. Second, the upper third of the ureter is more often injured than the middle and lower third. Third, associated injuries are frequently present. Fourth, CT scan and retrograde pyelography accurately identify ureteral injuries when performed in concert. Fifth, ureteroureterostomy, with or without indwelling stent, is the surgical procedure of choice of both trauma surgeons and urologists alike. Sixth and lastly, delay in diagnosis is associated with a worse prognosis.

## Competing interests

The authors declare that they have no competing interests.

## Authors' contributions

BMTP had overall responsibility for the study including conception, design and intellectual content, collection, analysis and interpretation of data; drafting and revision of the manuscript, figures and tables.

MPO participated in the collection, analysis and interpretation of data; revision of the manuscript, figures and tables.

JCGR participated in the analysis and interpretation of data; revision of the manuscript. Essential participation in the manuscript lay out, drafting figures and tables.

MLR participated in the collection, analysis and interpretation of data; revision of the manuscript, figures and tables.

DP participated in the collection of data.

ACM participated in the revision of the manuscript, figures and tables.

LRP participated in the revision of the manuscript, figures and tables.

MGM participated in the intellectual content; revision of the manuscript, figures and tables.

All authors read and approved the final manuscript.

## Supplementary Material

Additional file 1 **Ureteral Injuries Medical Literature Review**. The medical literature review table is organized by year of publication in descending order (2008 -1961). Authors, study design, objectives, incidence (demographics, type of injury, ureter injured portion), admission diagnostics (urine analysis, IVU, CT scan, RPG, intraoperative diagnosis), surgical technique and complications (early and late) were compiled [7-9,11,13,16,19-21,25,27-29,32-36,39,42,48,49,51-53,55-59,61,62,64,67,69,81-119]. Click here for file
