# Supplementary material for: A review of ureteral injuries after external trauma
Source: Scand J Trauma Resusc Emerg Med. 2010 Feb 3;18:6. doi: 10.1186/1757-7241-18-6 (PMC2830948; doi:10.1186/1757-7241-18-6)
Supplement: Additional file 1 — Ureteral Injuries Medical Literature Review. The medical literature review table is organized by year of publication in descending order (2008 -1961). Authors, study design, objectives, incidence (demographics, type of injury, ureter injured portion), admission diagnostics (urine analysis, IVU, CT scan, RPG, intraoperative diagnosis), surgical technique and complications (early and late) were compiled [7-9,11,13,16,19-21,25,27-29,32-36,39,42,48,49,51-53,55-59,61,62,64,67,69,81-119]. [file 1757-7241-18-6-S1.DOCX]

| Author’s | Type of Study | Incidence | Diagnosis  (Admission) | Surgical Approach (most common) | Complications (%) |
| --- | --- | --- | --- | --- | --- |
| Sonmez K[64] | CR | ♂ Age: 5/UL: 0% / PU: 100% | UA: N/P / Pre-Op IVU: N/P / IO 0% (missed) | U-U + stent 100% | 100 |
| Fraga GP[81] | R:8y(n=20) | ♂: 100% MA: 27/PUI: 100%/UL: 100%/MU: 50% | UAF: 50%  IO 90% | U-U + stent 60% | 55 |
| Madhok BM[82] | CR | ♀ A:15/UL/PU | IO: 100% | U-U + stent | 0 |
| Kunkle DA[83] | R:9y(n=40) | ♂: 99%/MA: 26.7/PUI: 92.5%/UL: 100%/DU: 34.3% PU: 34.3% | UAF: 55% / Pre-Op IVU: diagnostic in 11.4% / IO 82.9% | U-U 27.5%  U-U + stent 15% | 26.8 |
| Akay AF[84] | R:16y(n=21) | ♂: 76.1%/MA: 27.8/UL: 98%/PUI: 100%/DU: 50% | UAF: 71.4%  IO 100% | U-U + stent 80% | 4.7 |
| Best CD[11] | R:10y(n=57) | ♂: 96.5%/MA: 26/PUI: 94.5%/UL: 98%/MU: 36.8%/DU: 36.8% | UAF: 5%  Pre-Op IVU: diagnostic in 57% / IO 77% | U-U + stent 40% | 19 |
| Carver BS[85] | R:6y(n=12) | ♂: 91.6%/MA: 30.3/PUI: 94.5%/UL: 98%/ DU: 50% | UAF: 25% / No Pre-Op IVU / Pre-Op CT: 41.6%  IO 75% | U-U + stent 41.6% | 8.3 |
| Eliott SP[86] | R:25y(n=36) | ♂: N/A/MA: N/A/PUI: 91.6%/UL: 94.4%/PU: 39% | UAF: 25% / Pre-op/ Intra-Op IVP DxF: 60%  CT and RGP: 100%/IO: 64% | U-U + stent 38.8% | 18 |
| Hudolin[87] | R:3y(n=6) | Not informed | IO: 100% | U-U 80 % | N/A |
| Pearlstein DP[88] | CR | ♂ Age: 31/UL (duplicated)/MU | UA: 100% / CT: 100% | Stent | 0 |
| Perez-Brayfield MR[34] | R:40y(n=118) | ♂: Majority (N/A)/MA: Majority (N/A)/UL: 99.1% / PU: 43% | UAF: 14.8%  Pre-op/ Intra-Op IVP DxF: 41% / IO: 46.6% | U-U + stent 64.4% | 20.3 |
| Kennedy F[89] | CR | ♂: 100%/MA: 48/PUI: 100%/UL: 0%/MU: 50% | UA: N/A  Pre-Op IVU: diagnostic in 100% / IO 100% | U-U + stent 100% | 0 |
| Palmer LS[13] | R:10y(n=20) | ♂: 95%/MA: 28/PUI: 100%/UL: 100%/DU: 50% | UAF: 25%  Pre-Op IVU: diagnostic in 25% / IO 75% | U-U + stent 70% | 30 |
| Estevão-Costa[67] | CR | ♂ Age: 9/PUI: 0%/UL  PU | UA: not specified / Pre-Op IVU: not specified  No diagnosis in admission | appendix autotransplantation | 100 |
| Ghali AM[53] | R:5y(n=7)* | ♂: 42.8%/MA: N/A/PUI: 25%/UL: 85.7%/PU: 85.7% | UA: N/P / Delayed: 71.4%  IO: 14% / RGP: 85.7% | U-U + stent 57.1% | 57.1 |
| Powell MA[90] | CR(n=3) | ♂: 33.3%/MA: 32.3  PUI: 0%/UL: 100%/PU: 100% | UAF: 0% / Pre-Op IVU: N/A  IO 100% | U-U + stent 33.3% | 0 |
| Medina D[91] | R:5y(n=20) | ♂: 100%/MA: 29/PUI: 95%/UL: 100%/PU: 70.5 % | UA: N/A  Pre-op/ Intra-Op IVP DxF: 80% / IO: 85% | U-U + stent 60% | 29 |
| Mulligan JM[32] | R:5y(n=5) | ♂: 80%/MA:34.2/PUI: 0%/UL: 100%/PU: 100% | UAF: 20% / Pre-Op/ Intra-Op IVU: 0%  CT failed in 100% / IO 20% | Nephrectomy 40% | 80 |
| Azimuddin K[42] | R:8y(n=21) | ♂: 90%/MA: 28  PUI: 100%/UL: 95%/MU: 34% | UAF: 44%  Pre-op/ Intra-Op IVP DxF: 86% / IO: 95.2% | U-U + stent 76.1% | 20 |
| Velmahos GC[51] | R:1y(n=7) | ♂: N/A/MA: N/A/PUI: 100%/UL: N/A/Ureter portion: N/A | Not specified for ureteral injuries only | U-U 71.4% | 28.5 |
| Tucak A[92] | R:18m(n=11) | ♂: N/A/Age: N/A/PUI: 18%/Blast injuries: 82%/UL: N/A/MU: N/A | UA: N/P  Pre-op/ Intra-Op IVP N/P IO: 100% | UNC 63.6% | 33.3 |
| Marekovic Z[93] | R:30m(n=9) | ♂: N/A/Age: N/A/PUI: 100%/UL: 77.7%/MU: N/A | UA: N/P  Pre-op/ Intra-Op IVP N/P IO: N/A | UNC 72.2% | N/A |
| Velmahos GC[94] | R:6y(n=41) | ♂: 97.5%/MA: 29/PUI: 100%/UL: 100%/Ureter Portion injuried: N/A | UAF: 17.1%  IVP was accurate in 71.4% / IO: 82.1% | U-U + stent 80.5% | 26.8 |
| Kotkin L[28] | CR | ♂ Age: 12/UL/PU | UAF:100% IVP + RGP | U-U + stent | 0 |
| Soria Ruiz JS[95] | CR(n=2) | PUI: 50% | CT: 100% | U-U and appendix interposition | N/A |
| Vuckovic I[62] | R:34m(n=49)* | N/A | IO: 79.6% | U-U 32.1% | N/A |
| Brandes SB[96] | R:10y(n=12) | ♂: 91.6%/MA: 27/UL: 100%/MU: 58.3% | UAF: 45% / Intra-Op IVP DxF: 100%  IO: 91.6% | U-U + stent 83.3% | 25 |
| Wazzan W[97] | CR | ♂ Age: 19/UL/MU | UAF: 100% / IVP: N/A for early diagnosis  IO: 0% (delayed diagnosis) | Autotransplantation + Ureteropyelostomy | 100 |
| Boone TB[98] | R:10y(n=7) | ♂: N/A/MA: 18.2/UL: 85.7% | UAF: in 71.4% / IVP: N/A / IO: 43% | U-U 100% | 57 |
| Campbell[99] | R:27m(n=16) | ♂: 93.7%/MA: 32  PUI: 75%/UL: 87.5%/DU: 33.3% MU: 33.3% | UAF: 33.3% / IVP DxF: 66.6% / Pre-Op CT: 33.3%  IO 40% | U-U + stent 73.3% | 20 |
| Cofer BR[100] | CR | ♂ Age: 27/UL/PU | UA: 100% / IO: Missed (duplicated ureter) | PR and drainage | 100 |
| Seiler RK[69] | CR | ♂ Age: 6/UL/Blunt Trauma/PU | UA: 100% / Pre-Op IVP:N/A CT: 100% /IO: 100% | Ileal Interposition | 0 |
| Guerriero WG[101] | R:5y(n=49) | ♂: 91,8%/MA: N/A/PUI: 93.8/UL:N/A/PU: 45% | UA: N/A / Pre-Op IVP failed 11.9% / IO: 100% | U-U + stent 81.6% | 18.3 |
| Rober PE[57] | R:7y(n=16) | ♂: 93.7%/MA: 26/UL: 93.7%/MU: 50% | UAF: 28.5% / Pre-Op IVP diagnosis: 50% / IO: 50% | U-U + stent 62.5% | 25 |
| Presti JC Jr[8] | R:10y(n=18) | ♂: 94.4%/MA: 28/PUI: 88.8%/UL: 94.7%/PU: 58.8% | UAF: 31% / Pre-op/ Intra-Op IVP DxF: 36.3%  CT: 100% / IO: 66.6% | PR: 58% / U-U 32%  + stent 66.6% | 35.7 |
| Franco I[102] | R:9y(n=21) | ♂: 100%/MA: 27.5/UL: 100%/MU: 48% | UA: N/A / IVP failed in 61.9% / IO: 53.3% | U-U + stent 71% | 33.3 |
| Beamud-Gomez A[103] | R:17m(n=5) | ♂: 20%/MA: 4.8/UL:100%/PUI:0%/PU: 100% | UAF: 60% / IVP: N/A / IO: N/A | N/A | 20 |
| Cecconi RD[104] | CR | ♂ Age: 32/BL/DU | UAF: 100% / Pre-op IVP failed / IO: 100% | U-U + stent | 0 |
| Spirnak JP[59] | R:(n=12) | ♂: 75%/MA: 22.8/UL: 100%/PU: 37.5%/MU: 37.5% | UAF: 25% / Pre-op IVP failed in 50% / IO: 50% | U-U 37.5 % | 50 |
| Grizic AM[105] | R/P:7y(n=19)* | ♂: N/A/MA:N/A/UL: N/A/PUI: 63.1%/Ureteral injury: N/A | At time of presentation (not specified) or IO: 53% | N/A | N/A |
| Starinsky R[106] | CR(n=3) | ♂: 33.3%/MA: 25.2/UL: 100%/PUI: 0%/Ureteral injury: N/A | UAF: 100% / IVP: N/P  RGP: 100% / IO: 0% | Nephrectomy 33.3% | 100 |
| Cass AS[20] | R:12y(n=12) | ♂: 83%/Age Range: >20-59/UL: 100%/PU: 50% | UAF: 25%  IVP failed: 100% / IO: 20% | Expectant 80% | 33.3 |
| Cass AS[107] | R:21y(n=9) | ♂: 55.5%/MA: 42.5  UL: 90%/DU: 77.7% | UAF: 55.5% / IVP failed: 15% / IO: 33.3% | PR + ureterostomy: 33.3% / PR + nefrostomy 33.3% | 44.4 |
| Archbold JAA[61] | R:10y(n=8) | ♂: 100%/Age: N/A  UL: 100%/PU: 37.5%/DU: 37.5% | UA: N/A / IVP: N/A / IO: 87.5% | Expectant 50% (bruised ureter) | 25 |
| Pitts III, JC[48] | R:10y(n=18) | ♂: 83.3% / Age Range: 16-55 / UL: 100% / MU: 38.8% | UAF: 25% / Intra-Op IVP DxF: 27.7% / IO: 33.3% | U-U + stent 88.8% | 38.8 |
| Kirchner FK[7] | CR | ♂ Age: 22/BL/MU | UAF: 0% / IO diagnosis: 100% | U-U + stent | 0 |
| Drago JR[108] | CR | ♂ Age: 43/BL/DU | UAF: 0% / IVP failed: 0% / IO: 100% | U-U + stent 100% | 100 |
| Cass AS[16] | R:17y(n=11) | N/A | UAF: 14.7% / IVP failed: 100% / IO: 54.5% | Expectant 80% | 33.3 |
| Ambiavagar R[39] | R:7y(n=6) | ♂: 100%/MA: 14.8/UL: 100%/PUI:0%/PU: 100% | UAF: 50% / IO: 20% | U-U 50% | 17 |
| Laberge I[109] | R:N/A(n=6) | ♂ Age range:3-35/UL: 100%/Proximal+ MU 100% | UAF: 50% / IVP failed: 30%  RGP: 100% / IO: Confirmed | U-U + stent 100% | 40 |
| McDonald WB[21] | CR | ♂ Age: 14/UL/MU | UA: 100% / IVP: no contrast extravasation IO | PR | 0 |
| McGinty DM[56] | R:10y(n=9) | “Young ♂s” 90%/UL: 100%/PU: 100% | All 9 were not diagnosed until after the exploratory laparotomy or for several days after injury  IVP 100% reliable on 9 cases of delayed diagnosis | Nephrectomy 44.4% | 100 |
| Liroff AS[110] | R:5y(n=20) | ♂: N/A/Age: 33/UL: N/A/MU: 55% | UAF: 30.7% / Pre Intra-Op IVP DxF: 10% / IO: 40% | U-U + stent 65% | 30 |
| Stutzman RE[25] | R:3y(n=21) | ♂: N/A/Age: 21/UL: 95.4/DU: 59% | UA: N/A / Pre/ Intra-Op IVP diagnosis N/A / IO: N/A | U-U 50% | 41 |
| Rabinowitz R[111] | CR | ♂ Age: 9/UL/PU | UA: N/A / Pre/ Intra-Op IVU: N/A / IO: 0%  (delayed diagnosis) | U-U + stent | 100 |
| Evans RA[27] | R:7y(n=13) | ♂: N/A/ Age: N/A/UL: 93.7%/PUI: 53.2%/PU Only | UAF: 53.4% / Intra-Op IVP DxF: 14.2% / IO: 40% | PR 50% | 50 |
| Bright[35] | R:10y(n=59) | ♂: N/A/ Age Range: N/A/UL: 100%/PUI: 96.6%/MU: 40% | UAF: 37.2% / Pre-Op IVP DxF: 45.8% / IO: 95% | U-U + stent 47.4% | 18.6 |
| Heath AD[112] | CR | ♂: N/A/ Age Range: 3/UL: 0%/PUI: 0% / PU: 100% | UA: N/P / Pre-Op IVP DxF: 0% / IO: 100% | U-U + stent 100% | 0 |
| Lankford R[55] | R:3y(n=10) | ♂: 90%/MA: 27/UL: 100%/PU: 70% | UAF: 0% / Pre-op IVP: 100% / IO: confirmed | U-U + stent 60% | 30 |
| Reznichek RC[29] | CR(n=2) | ♂: 50%/MA: 12.5/UL: 100%/PUI: 0%/Proximal and DU | UAF: 0% / Pre-op IVP: 100% / IO: 100% | U-U 100% | 0 |
| Johnson JM[113] | CR | ♂: N/A/Age: 3/PUI:0%/UL: 0%/PU | UAF: 0% / Pre-op IVP failed in: 50% / IO: 50% | U-U + stent | 100 |
| Fisher S[52] | R:1y(n=9) | ♂: N/A/Age Range: N/A/UL: 100%/Injury Location: N/A | N/A | U-U 77.7% | 88.8 |
| Del Villar RG[114] | R:10y(n=6) | ♂: N/A/MA: 41.5/UL: 100%/PUI: 33.3%/PU: 83.3 % | UAF: 40% / IVP failed in: 25% / IO: 50% | U-U 66.6% | 66.6 |
| Beckly DE[33] | CR | ♂ Age: 9/UL/ | UAF: 0% / Pre-op IVP failed in: 0% / RGP: 100% / IO: 66.6% | N/A | 0 |
| Slater RB[115] | CR | ♂ Age: 17/UL | N/A | U-U + stent | 0 |
| Carlton CE Jr[36] | R:14y(n=39) | ♂: N/A/Age: N/A/UL: N/A | UAF: 10% / Pre-op IVP failed in:14% / IO: confirmed | N/A | N/A |
| Salvatierra O Jr[58] | R:4y(n=11) | ♂: 90.9%/MA: 27.5/UL: 100%/PU: 45.4% | UA:N/A / Pre-op IVP: 100% (When performed)  RGP: 100% / IO: 81.8% | U-U + stent 72.2% | 18.1 |
| Walker JA[49] | R:8y(n=27) | ♂: N/A/MA: N/A/UL: 100%/PUI: 88.8%/MU: 44.4% | UAF: 29.6% / Pre-op IVP: 87.5% (When performed) / IO:N/A | N/A | 40.7 |
| Halverstadt BD[116] | Case report | ♂: 0%/Age: 4/UL | UA: 100% / Pre-op IVP: 100% / RGP: 100% / IO: confirmed | U-U + stent | 0 |
| Ainsworth T[117] | Case report | ♂: 0%/MA: 58/UL: 0%/PUI: 0%/PU | UAF: 100% / RGP: 100% / IO: 100% | U-U + stent | 0 |
| Fruchtman B[118] | CR(n=2) | ♂: 100%/MA: 11/UL: 100% | UAF: in 0% / Pre-op IVP: 100% / IO: confirmed | U-U + stent | 0 |
| Stone HH[119] | R:N/A(n=8) | ♂: N/A/Age: N/A/PUI: 87.5%/UL: 100%/PU: 62.5% | UAF: 12.5% / IVP and RPG: 100% / IO: 0% | U-U 50% | 100 |
| Pumphrey JD[19] | R:20y(n=6) | ♂: N/A/Age: N/A/UL: 75%/PU: 59% | UA: N/A / IVP: N/A / IO: 25% | N/A | N/A |
| Zufall R[9] | CR | ♂ Age: 5/UL/PU | UAF: 100%/ IVP: N/A /Delayed diagnosis | U-U +stent | 100 |

R: Retrospective Study. CR: Case Report. MA: Mean Age, UL: Unilateral. BL: Bilateral. PU: Proximal Ureter. DU: Distal Ureter. MU: Mid- ureteral. UA: Admission urinanalysis. UAF: Urinanalysis Diagnostic Failure. DxF: Diagnostic failure. U-U: Uretero-ureterostomy. PR: Primary repair. PUI: Penetrating Ureteral Injuries/ IVU: Intra venous Urography/ IVP: Intra venous Pyelography/ RGP: Retrograde Pyelography/ UPJ: Ureteropelvic Junction/ GSW: Gun Shot Wounds/ IO: Intra-operative diagnosis

* Attention was given only for ureteral injuries due to external trauma
